# Leptin is associated with vascular endothelial function in overweight patients with type 2 diabetes

**DOI:** 10.1186/1475-2840-13-10

**Published:** 2014-01-10

**Authors:** Tomoaki Morioka, Masanori Emoto, Yuko Yamazaki, Naoya Kawano, Satoshi Imamura, Ryutaro Numaguchi, Hiromi Urata, Koka Motoyama, Katsuhito Mori, Shinya Fukumoto, Hidenori Koyama, Tetsuo Shoji, Masaaki Inaba

**Affiliations:** 1 Departments of Metabolism, Endocrinology and Molecular Medicine, 1-4-3, Asahi-machi, Abeno-ku, Osaka 545-8585, Japan; 2 Department of Internal Medicine, Division of Endocrinology and Metabolism, Hyogo College of Medicine, 1-1, Mukogawa-cho, Nishinomiya, Hyogo 663-8501, Japan; 3 Department of Geriatrics and Vascular Medicine, Osaka City University Graduate School of Medicine, 1-4-3, Asahi-machi, Abeno-ku, Osaka 545-8585, Japan

**Keywords:** Leptin, Endothelial function, Overweight, Type 2 diabetes

## Abstract

**Background:**

The adipocyte-derived hormone leptin plays a key role in the regulation of appetite and body weight. Recent studies have suggested that leptin is also involved in the pathogenesis of obesity-related atherosclerosis and cardiovascular disease. In this study, we investigated the association of plasma leptin levels with vascular endothelial function in lean and overweight patients with type 2 diabetes.

**Methods:**

One hundred seventy-one type 2 diabetic patients, of which 85 were overweight (body mass index (BMI) ≥ 25 kg/m^2^), were enrolled in this cross-sectional study. Plasma leptin concentrations were measured by enzyme-linked immunosorbent assay. Flow-mediated dilatation (FMD) of the brachial artery was measured to evaluate vascular endothelial function using ultrasound.

**Results:**

No significant difference in FMD was found between the lean and overweight groups (7.0 ± 3.8% and 6.5 ± 3.6%, respectively; *p* = 0.354). FMD was negatively correlated with age (*r* = −0.371, *p* < 0.001) and serum creatinine levels (*r* = −0.236, *p* = 0.030), but positively correlated with BMI (*r* = 0.330, *p* = 0.002) and plasma leptin levels (*r* = 0.290, *p* = 0.007) in the overweight group. FMD was not associated with any parameters in the lean group. Multiple regression analysis including possible atherosclerotic risk factors revealed that the plasma leptin level (*β* = 0.427, *p* = 0.013) was independently associated with FMD in the overweight group (*R*^*2*^ = 0.310, *p* = 0.025), but not the lean group.

**Conclusion:**

Plasma leptin levels are associated with vascular endothelial function in overweight patients with type 2 diabetes.

## Background

Obesity is a serious health problem worldwide and is associated with established cardiovascular risk factors including hypertension, dyslipidemia, insulin resistance, and diabetes [1]. Adipose tissue exerts endocrine and immune functions by releasing bioactive mediators and adipocytokines such as leptin, adiponectin, resistin, tumor necrosis factor-α, interleukin-6, and monocyte chemotactic protein-1 [2]. Leptin was one of the first adipocytokines identified and has been extensively investigated. Leptin is produced predominantly by adipose tissue and plays a pivotal role in the regulation of appetite and body weight. Plasma leptin levels are markedly elevated in obese individuals, and leptin receptors are widely distributed in peripheral tissue including the cardiovascular system [3]. Therefore, hyperleptinemia may be one potential mechanism linking obesity to atherosclerotic cardiovascular disease. Recent *in vitro* and *in vivo* studies have indicated that, in addition to its major roles in energy metabolism, leptin is also involved in the pathophysiology of atherosclerosis [1,2,4]. Moreover, several clinical studies have shown that the plasma leptin level is an independent predictor of incident coronary artery disease [5,6].

The integrated effect of leptin on vascular endothelial function, a key factor for the initiation and development of atherosclerotic vascular damage [7], remains to be elucidated, since contradictory findings have been reported from experimental and clinical studies. For example, leptin was found to induce endothelium-dependent vascular relaxation by stimulating nitric oxide (NO) in studies using isolated aortic rings of rats [8,9]. Leptin infusion also caused vasodilatation of the brachial artery [10] and coronary artery [11] in non-obese, healthy human subjects. Nonetheless, in pathological conditions such as obesity or metabolic syndrome (MetS), resistance to leptin’s vasodilatory effect has been observed in both animal [12-14] and human studies [15,16]. Obesity and hyperleptinemia caused by diet lead to impaired leptin-induced NO and cyclic guanosine monophosphate production in the aortic wall of rats [12] and in aortic endothelial cells of mice [13]. Knudson et al. [14] performed intracoronary leptin dose–response experiments in anesthetized dogs and found that obese levels of coronary plasma leptin (mean 81 ng/ml) attenuated acetylcholine-induced coronary artery relaxation, whereas normal physiologic levels of leptin (approximately 4 ng/ml) had no effect. Human studies also demonstrated that the serum leptin level is inversely associated with adenosine-stimulated myocardial blood flow in young obese men [15], and with forearm endothelium-dependent vasodilatation (EDV) in the elderly [16]. Nevertheless, controversy persists over whether an independent clinical association between leptin and vascular endothelial function exists, since the associations in the abovementioned studies were no longer present or were attenuated after adjustment for body mass index (BMI) in humans [15,16].

To our knowledge, no study has thus far investigated whether leptin plays a role in vascular endothelial function in patients with type 2 diabetes (T2D), in whom vasodilation mediated by endothelium-derived NO is impaired [17]. Therefore, the aim of the present study was to clarify the cross-sectional association between plasma leptin levels and vascular endothelial function, assessed by flow-mediated dilatation (FMD) of the brachial artery using ultrasound, in patients with T2D.

## Methods

### Subjects

We consecutively enrolled 171 subjects with T2D (89 men and 82 women) who were admitted to the Diabetes Center of the Osaka City University Hospital between January 2009 and September 2011. T2D was diagnosed on the basis of the criteria of the American Diabetes Association [18]. Smokers were defined as current or past smokers in our analyses. Subjects were divided into either the lean (BMI < 25 kg/m^2^) or overweight (BMI 25 ≥ kg/m^2^) group for analyses. Subjects with type 1 diabetes, other types of diabetes, or renal impairment with a serum creatinine level ≥ 1.1 mg/dL, which is the upper limit of the normal range in our laboratory, were excluded from the present study. All subjects provided written informed consent, and the ethical review board of our institution approved this study protocol.

### Physical and laboratory analyses

Blood pressure was determined by the conventional cuff method using a mercury sphygmomanometer after subjects rested for at least 15 min. Blood samples were drawn after an overnight fast and biochemical parameters were analyzed by a standard laboratory method as previously described [19]. Immunoreactive insulin was measured for subjects not receiving insulin therapy (n = 108). Plasma leptin levels were measured using enzyme-linked immunosorbent assay kits (R & D Systems, Minneapolis, MN). The minimum detectable level of leptin was 0.16 ng/mL, and the intra- and inter-assay coefficients of variation were 3.2% and 3.5%, respectively [20].

### Measurement of FMD

We measured FMD of the brachial artery according to the International Brachial Artery Reactivity Task Force guidelines [21] and the Japanese guidelines of the Vascular Failure Workshop Group [22] using a novel ultrasound system equipped with an edge-tracking system for 2D imaging and a pulsed Doppler flow velocimeter for automatic measurement (UNEXEF; Unex Co. Ltd., Nagoya, Japan), as we [23] and others [24,25] previously described. In brief, the diameter of the brachial artery at rest was measured in the cubital region. Subsequently, the cuff was inflated to 50 mmHg above systolic blood pressure (SBP) for 5 min and then deflated. The diameter of the artery was monitored continuously at the same point, and the maximum dilatation from 45–60 s after deflation was recorded. Following FMD measurement, endothelium-independent nitroglycerin-mediated dilatation (NMD) was also measured. After a 15-min rest for vessel recovery, sublingual nitroglycerin (75 μg) was administered, and the maximum dilatation of the brachial artery at the same point was measured during at least 1 min after the initiation of maximum dilatation. FMD and NMD were calculated as follows: FMD or NMD (%) = (maximum diameter – diameter at rest) × 100/diameter at rest.

### Statistical analysis

Statistical analyses were performed using the JMP® 9 software (SAS Institute Inc., Cary, NC). All results were expressed as mean ± standard deviation (SD) or median (interquartile range) as appropriate. Student’s *t*-test, Wilcoxon rank-sum test, or χ^2^-test was performed where appropriate for comparisons between the lean and overweight groups. Simple linear regression analyses and multiple regression analyses were performed to evaluate the relationships between FMD and various clinical parameters including plasma leptin level. Skewed parameters such as immunoreactive insulin, triglycerides, and plasma leptin levels were logarithmically transformed before regression analyses. In multiple regression analyses, FMD was the dependent variable and plasma leptin level as well as age; sex; BMI; waist circumference; SBP; creatinine level; HbA1c; triglyceride level; high-density lipoprotein-cholesterol (HDL-C) level; low-density lipoprotein-cholesterol (LDL-C) level; smoking status; and treatment with insulin, statins, or angiotensin-II receptor blockers or angiotensin-converting enzyme inhibitors (ARB/ACEI) were independent variables. A *p*-value < 0.05 was considered significant.

## Results

### Clinical characteristics of the subjects

The clinical characteristics of the lean and overweight groups as well as the total population are shown in Table 1. The mean ± SD for the age of all subjects was 63 ± 10 years (range, 36–86 years). Twenty subjects were treated with dietary therapy alone, 88 with oral hypoglycemic agents, 39 with insulin, and 24 with a combination of insulin and oral hypoglycemic agents. Seventy-one subjects were treated with 3-hydroxy-3-methyl-glutaryl-CoA reductase inhibitors (statins) and 72, with ARB/ACEI. Overweight subjects were significantly younger than the lean subjects. As expected, overweight subjects had higher diastolic blood pressure (DBP), plasma insulin, insulin resistance index by homeostasis model assessment (HOMA-R), serum triglycerides, uric acid levels and lower high-density lipoprotein cholesterol (HDL-C) levels than the lean subjects. There were no significant differences among the lean and overweight groups for frequency of smoking, insulin use, ARB/ACEI, or statin treatment. The median plasma leptin level for all subjects was 4.1 (2.0–8.0) ng/mL. Plasma leptin levels in the overweight group were also significantly higher than that in the lean group (5.9 (3.2–9.8) vs. 2.5 (1.2–4.8) ng/mL, *p* < 0.001). Mean FMD for the total population was 6.8 ± 3.7% (range, 0.7–18.5), and no significant differences between overweight and lean subjects was found (6.5 ± 3.6% vs. 7.0 ± 3.8%, *p* = 0.354). Mean NMD was also not statistically different between the two groups (15.5 ± 7.3% vs. 14.6 ± 6.6%, *p* = 0.424).

**Table 1:** Clinical characteristics, plasma leptin levels, FMD and NMD of all, lean, and overweight subjects

		**All**	**Lean**	**Overweight**	***p***
N	(Male/female)	171 (89/82)	86 (40/46)	85 (43/42)	0.704
Age	(years)	63 ± 10	66 ± 9	60 ± 11	<0.001
BMI	(kg/m^2^)	24.8 ± 4.9	21.3 ± 2.3	28.3 ± 4.4	<0.001
Waist	(cm)	87 ± 10	80 ± 7	94 ± 8	<0.001
Waist-to-hip ratio		0.93 ± 0.05	0.92 ± 0.05	0.96 ± 0.05	<0.001
SBP	(mmHg)	129 ± 17	129 ± 17	130 ± 17	0.774
DBP	(mmHg)	75 ± 9	73 ± 8	77 ± 9	0.006
Smoker	n (%)	72 (42.1)	35 (40.7)	37 (43.5)	0.708
Insulin	n (%)	63 (36.8)	37 (43.0)	26 (30.6)	0.091
ARB/ACEI	n (%)	72 (42.1)	40 (46.5)	32 (37.6)	0.240
Statins	n (%)	71 (41.5)	37 (43.0)	34 (40.0)	0.688
Glucose	(mg/dL)	127 ± 35	129 ± 35	125 ± 35	0.524
HbA1c	(NGSP,%)	8.7 ± 1.6	8.8 ± 1.7	8.6 ± 1.4	0.399
IRI	(μU/mL)	6.6 (4.4–9.7)	5.3 (3.1–8.2)	8.1 (5.2–11.8)	<0.001
HOMA-R		2.0 (1.3–3.0)	1.4 (0.9–2.5)	2.3 (1.4–3.4)	0.002
TG	(mg/dL)	106 (81–136)	95 (72–134)	113 (91–137)	0.035
HDL-C	(mg/dL)	46 ± 13	49 ± 14	43 ± 10	0.003
LDL-C	(mg/dL)	109 ± 37	110 ± 38	108 ± 37	0.728
Cre	(mg/dL)	0.8 ± 0.2	0.7 ± 0.2	0.8 ± 0.2	0.365
Uric acid	(mg/dL)	5.7 ± 1.4	5.4 ± 1.4	5.9 ± 1.4	0.010
Leptin	(ng/mL)	4.1 (2.0–8.0)	2.5 (1.2–4.8)	5.9 (3.2–9.8)	<0.001
FMD	(%)	6.8 ± 3.7	7.0 ± 3.8	6.5 ± 3.6	0.354
NMD	(%)	15.0 ± 7.0	15.5 ± 7.3	14.6 ± 6.6	0.424

Data are expressed as mean ± SD, median (interquartile), or N (%) as appropriate. BMI, body mass index; Waist, waist circumference; SBP, systolic blood pressure; DBP, diastolic blood pressure; smoker, past and current smokers; insulin, frequency of subjects treated with insulin; ARB/ACEI, frequency of subjects treated with angiotensin II receptor antagonists or ACE inhibitors; statins; frequency of subjects treated with stains; IRI, immunoreactive insulin; HOMA-R, insulin resistance index by homeostasis model assessment; TG, triglycerides; HDL-C, high-density lipoprotein cholesterol; LDL-C, low-density lipoprotein cholesterol; Cre, serum creatinine; FMD, endothelium-dependent, flow-mediated dilatation of the brachial artery; NMD, endothelium-independent, nitroglycerin-mediated dilatation of the brachial artery.

### Association between plasma leptin levels and FMD

**Figure 1:**
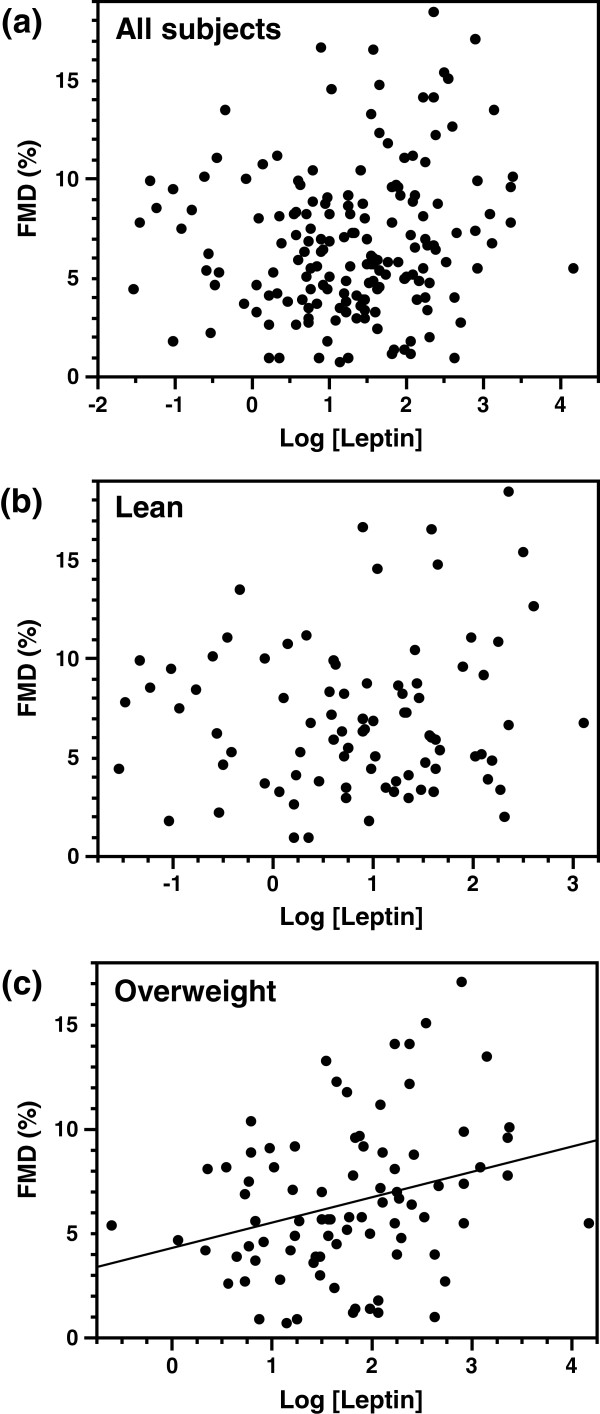
**Association of plasma leptin levels with the flow-mediated dilatation (FMD) of the brachial artery in (a) all subjects, (b) the lean group, or (c) the overweight group.** Plasma leptin levels were positively correlated with FMD in the overweight group, but not the lean group or the total population.

First, we examined the association between FMD and plasma leptin levels or clinical risk factors for atherosclerosis by simple linear regression analyses for the total population and then for lean and overweight subjects separately (Table 2). In all subjects, age and creatinine level were significantly and negatively correlated with FMD. In the lean group, none of the parameters were significantly correlated with FMD. Age, waist-to-hip ratio and creatinine level were negatively correlated with FMD, and BMI was positively correlated with FMD in the overweight group. Plasma leptin levels exhibited a significant, positive correlation with FMD in the overweight group, but not the lean group or the total population (Table 2 and Figure 1). Consistent with previous reports [23,26], NMD was negatively associated with age, waist circumference, waist-to-hip ratio, SBP, and creatinine level in all, lean and/or overweight subjects. However, plasma leptin levels were not associated with NMD in either the lean or overweight group, which is in contrast to the relationship we found between plasma leptin levels and FMD. In subjects not receiving insulin therapy (n = 108), plasma leptin levels were also significantly correlated to FMD (*r* = 0.337, *p* = 0.009), but not to NMD (*r* = 0.224, *p* = 0.088), whereas insulin resistance assessed by HOMA-R was not correlated to FMD (*r* = −0.023, *p* = 0.873) or NMD (*r* = −0.124, *p* = 0.382), in the overweight group (n = 59).

**Table 2:** Correlations between FMD, NMD, and clinical variables in subjects with type 2 diabetes

	**FMD**						**NMD**					
	**All subjects**		**Lean**		**Overweight**		**All subjects**		**Lean**		**Overweight**	
	***r***	***p***	***r***	***p***	***r***	***p***	***r***	***p***	***r***	***p***	***r***	***p***
Age	−0.175	0.022	−0.007	0.948	−0.371	<0.001	−0.425	<0.001	−0.382	<0.001	−0.545	<0.001
BMI	0.101	0.191	0.034	0.756	0.330	0.002	−0.044	0.572	−0.192	0.077	0.111	0.313
Waist	0.014	0.852	0.005	0.967	0.155	0.159	−0.169	0.028	−0.287	0.008	−0.051	0.648
Waist-to-hip ratio	−0.134	0.083	0.067	0.545	−0.285	0.009	−0.220	0.004	−0.200	0.067	−0.225	0.040
SBP	−0.123	0.110	−0.054	0.622	−0.193	0.076	−0.315	<0.001	−0.405	<0.001	−0.213	0.051
DBP	0.032	0.676	0.172	0.113	−0.059	0.593	−0.005	0.946	−0.053	0.627	0.065	0.555
Cre	−0.168	0.028	−0.090	0.409	−0.236	0.030	−0.161	0.036	−0.084	0.441	−0.235	0.031
Glucose	−0.050	0.519	−0.117	0.284	0.015	0.893	−0.069	0.370	−0.033	0.765	−0.118	0.284
HbA1c	−0.147	0.055	−0.174	0.108	−0.125	0.253	0.012	0.875	0.029	0.788	−0.021	0.846
Log [IRI]	−0.049	0.622	−0.009	0.954	0.035	0.799	−0.069	0.487	−0.068	0.642	−0.019	0.892
Log [HOMA-R]	−0.047	0.639	0.007	0.961	0.013	0.925	−0.125	0.206	−0.140	0.338	−0.071	0.608
Log [TG]	0.025	0.746	0.024	0.829	0.056	0.614	0.024	0.756	0.082	0.455	−0.037	0.739
HDL-C	0.025	0.748	−0.016	0.881	0.045	0.682	−0.098	0.203	−0.116	0.286	−0.115	0.295
LDL-C	−0.004	0.962	−0.108	0.324	0.105	0.339	0.135	0.079	0.176	0.106	0.085	0.442
Uric acid	0.069	0.369	0.024	0.827	0.147	0.179	−0.016	0.838	0.018	0.873	−0.027	0.809
Log [Leptin]	0.136	0.077	0.107	0.329	0.290	0.007	−0.0003	0.997	−0.0001	0.999	0.070	0.522

*r*, correlation coefficient by simple regression analysis. Abbreviations are the same as Table 1.

**Figure 2:**
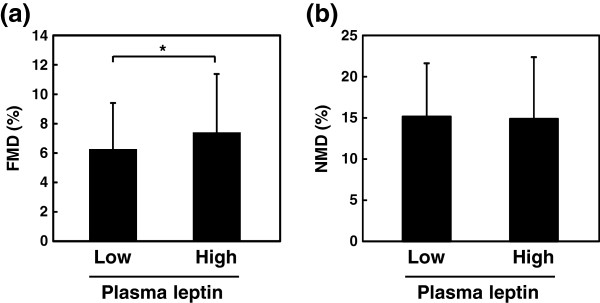
**Comparison of FMD (a) or NMD (b) between high and low plasma leptin groups.** *, p < 0.05. The subjects with higher leptin levels exhibited significantly higher FMD, but not NMD, than subjects with lower leptin levels.

In keeping with the positive correlation between plasma leptin and FMD, subjects with plasma leptin above the median (4.1 ng/mL) exhibited grater FMD than those with plasma leptin below the median (7.3 ± 4.0 vs. 6.2 ± 3.2%, *p* = 0.042) (Figure 2). Mean NMD was not different between subjects with plasma leptin above and below the median (14.9 ± 7.5 and 15.2 ± 6.5%, respectively, *p* = 0.799).

Since FMD could be affected by various clinical factors and medication use for diabetes, hypertension, or dyslipidemia [27,28], multiple regression analyses were performed in order to identify the impact of plasma leptin on FMD while controlling for important confounders in all, lean and overweight subjects, as described in the Statistical analysis section (Table 3). Among all independent variables, only plasma leptin levels (*β* = 0.427, *p* = 0.013), but not BMI (*β* = 0.107, *p* = 0.648) or waist circumference (*β* = −0.186, *p* = 0.362), significantly contributed to FMD in the overweight group. However, in the lean group, no parameters were significantly associated with FMD (*R*^*2*^ = 0.111, *p* = 0.885).

**Table 3:** Multivariate analyses of the determinants for FMD and NMD in subjects with type 2 diabetes

	**FMD**						**NMD**					
	**All subjects**		**Lean**		**Overweight**		**All subjects**		**Lean**		**Overweight**	
	***β***	***p***	***β***	***p***	***β***	***p***	***β***	***p***	***β***	***p***	***β***	***p***
Age (years)	−0.178	0.081	−0.058	0.692	−0.246	0.096	−0.453	<0.001	−0.149	0.227	−0.724	<0.001
Sex (male = 1)	−0.014	0.917	−0.195	0.346	0.167	0.379	0.116	0.310	0.214	0.217	0.028	0.863
BMI (kg/m^2^)	0.035	0.860	−0.037	0.871	0.107	0.648	−0.150	0.374	−0.250	0.196	−0.346	0.089
Waist (cm)	−0.150	0.403	0.048	0.807	−0.186	0.362	−0.366	0.018	−0.237	0.157	−0.131	0.457
SBP (mmHg)	−0.094	0.271	−0.064	0.643	−0.220	0.069	−0.241	0.001	−0.378	0.002	−0.203	0.053
Cre (mg/dL)	−0.046	0.644	0.054	0.732	−0.031	0.824	−0.076	0.375	0.010	0.941	−0.073	0.548
HbA1c (%)	−0.186	0.034	−0.155	0.314	−0.138	0.207	−0.009	0.907	0.011	0.931	−0.006	0.953
Log [TG (mg/dL)]	0.000	0.999	−0.035	0.836	0.110	0.355	−0.098	0.231	−0.057	0.691	−0.096	0.350
HDL-C (md/dL)	0.097	0.325	0.079	0.625	0.046	0.703	−0.125	0.134	−0.078	0.564	−0.233	0.027
LDL-C (mg/dL)	−0.051	0.554	−0.107	0.432	0.014	0.912	−0.050	0.494	0.045	0.694	−0.189	0.087
Smoking (yes = 1)	−0.080	0.378	−0.053	0.702	−0.049	0.712	0.026	0.732	0.064	0.580	−0.039	0.730
Insulin (yes = 1)	−0.050	0.572	−0.175	0.236	0.056	0.646	−0.073	0.338	−0.155	0.210	−0.066	0.530
Statins (yes = 1)	0.008	0.915	0.037	0.768	−0.035	0.747	−0.030	0.652	0.008	0.942	−0.032	0.727
ARB/ACEI (yes = 1)	−0.104	0.209	−0.069	0.576	−0.171	0.151	−0.040	0.572	−0.020	0.846	−0.019	0.851
Log [Leptin (ng/mL)]	0.158	0.246	0.038	0.859	0.427	0.013	0.341	0.004	0.462	0.011	0.318	0.031
R^2^	0.132	0.094	0.111	0.885	0.310	0.025	0.372	<0.001	0.376	0.002	0.485	<0.001

*β*, standard correlation coefficient by multiple regression analysis; *R*^*2*^, multiple coefficients of determination. Abbreviations are the same as Table 1.

## Discussion

The present study demonstrated that plasma leptin levels are positively related to vascular endothelial function in overweight T2D patients, but not in lean patients. Of importance, plasma leptin level was a significant contributing factor to FMD, independent of BMI, blood pressure and other traditional cardiovascular risk factors, in overweight T2D patients. Our findings suggest that leptin exerts its positive and vasodilator effect on endothelial function in overweight diabetic patients with an elevated risk for cardiovascular diseases.

A number of clinical studies have investigated the association between plasma leptin levels and vascular endothelial function [16,29-31]. Plasma leptin levels were reported to be negatively associated with EDV measured by forearm plethysmography in the elderly subjects [16], and with FMD of the brachial artery in patients with nonalcoholic fatty disease [29] and polycystic ovarian syndrome [30]. However, the association of plasma leptin with vascular endothelial function was not independent of the other variables including BMI in these studies. Plasma leptin-to adiponectin ratio was also shown to be negatively associated with FMD in healthy elderly subjects [31]. In the other studies, plasma leptin levels were not associated with FMD of the brachial artery [32-37]. FMD was evaluated after weight reduction by low-calorie diet for obese subjects in several studies [38-41]. Increased FMD after weight loss was negatively [38], positively [39], or not [40,41] associated with change in plasma leptin levels. These previous cross-sectional and interventional studies demonstrate inconsistent results on the relationship between leptin and endothelial function, possibly because the study subjects or the method used to estimate endothelial function differs. The findings of our study contrast with those of these previous studies in that the association of leptin levels with FMD was independent of confounding cardiovascular risk factors such as age, BMI, SBP, and lipids in overweight T2D patients. In addition, moderately elevated plasma leptin levels unexpectedly exhibited a positive relation with FMD in overweight patients with T2D in this study. These patients are also generally at a high risk for atherosclerosis and cardiovascular disease.

Functional leptin receptors are expressed in vascular endothelium [9]. Several experimental studies showed that acutely administered leptin induces endothelium-dependent vasorelaxation by stimulating the release of endothelial NO or the endothelium-derived hyperpolarizing factor (EDHF) in rats [8,9,42], or by neuronal NO synthase in mice [43]. Endothelium-independent vasodilation by leptin was also identified in the saphenous vein and internal mammary artery *ex vivo* in humans with coronary artery disease [44]. A direct vasodilator effect of acute leptin infusion has also been investigated in several human studies. Nakagawa et al. [10] reported forearm vasodilatation by intra-arterial infusion of leptin, and coronary artery vasodilation by intra-coronary leptin infusion in a separate study [11]. The leptin-induced vasodilatation was independent of NO and possible involvement of other vasoactive agents such as the EDHF or prostacyclin was suggested in those human studies [10,11]. Brook et al. [45] demonstrated that brachial FMD increased 2 hours following subcutaneous injection of recombinant human leptin without altering blood pressure in non-obese adults. These *in vitro* and *in vivo* data indicate a direct vasodilator effect of leptin on the endothelium through endothelial NO or other factors, and this could support our finding, at least in part, that an independent and positive association exists between plasma leptin levels and FMD in overweight subjects.

On the other hand, a number of studies have shown that leptin regulates the sympathetic nervous system, endothelin-1 production, and renin-angiotensin system [4,46], all of which contribute to vasoconstriction and may counteract the depressor effect of leptin on vascular function. Moreover, a large number of studies have indicated that leptin regulates immune function and cytokine secretion, upregulates C-reactive protein production, and increases oxidative stress in endothelial cells, all of which promote the pathophysiology of atherogenesis including endothelial dysfunction [1,4,47,48]. Indeed, obesity or long-term hyperleptinemia was shown to reduce NO bioavailability of the aortic endothelium mice [13] and rats [12], and to attenuate NO-dependent vasodilation of the coronary artery in dogs [14]. Schinzari et al. [49] demonstrated in human study that leptin infusion enhanced EDV in lean subjects, but not in patients with obesity-related MetS. High leptin concentrations were also reported to be associated with impaired EDV measured by forearm plethysmography [16] and inversely with adenosine-stimulated coronary blood flow [15] in human subjects, but those associations were not independent of BMI and/or insulin levels. These experimental and clinical studies indicate that the NO-dependent vasodilatory effects of leptin become impaired, and by this mechanism, leptin may contribute to endothelial dysfunction and the progression of atherosclerosis in patients with obesity and/or MetS. These studies imply that the selective leptin resistance seen in obesity may not be limited to appetite and body weight control, but may involve the hemodynamic actions of leptin, thus leading to the pro-atherogenic effects of leptin on vascularture [50]. However, to date, no previous study has demonstrated an independent association of leptin with brachial artery FMD assessed by ultrasound, in human subjects with obesity and/or MetS.

To our knowledge, this is the first study to explore the association of leptin with vascular endothelial function in patients with T2D. We found an independent and positive association between plasma leptin levels and FMD in overweight (BMI ≥ 25 kg/m^2^), but not lean subjects, even after adjustment for other confounders including age, BMI, SBP, HbA1c, and lipid levels. The positive association between plasma leptin levels and FMD in patients with diabetes is contrary to previous studies that show an association between hyperleptinemia and impaired endothelial function in patients with obesity and/or MetS. There are several possible explanations for this discrepancy. First, the degree of obesity in our Japanese patients was much less than that of the studies performed in European countries, which demonstrated a resistance to leptin-induced vasoreactivity in human subjects with obesity or MetS [15,49]. BMI and plasma leptin levels were 33.6 kg/m^2^ and 10.3 ng/mL, respectively, in obese subjects from the study by Sundell and colleagues [15], and 38 kg/m^2^ and 21.2 ng/mL, respectively, in subjects with MetS in the study by Schinzari and colleagues [49]. These BMI and leptin levels are much higher than those of our overweight subjects (BMI, 28.4 kg/m^2^; leptin levels, 6.0 ng/mL). Moreover, the plasma leptin levels of our overweight subjects were similar to those of the healthy [15] and control subjects [49], at 4.3 ng/mL and 8.7 ng/mL, respectively, in two studies from Europe. Therefore, the vasodilator effect of leptin could have still been activated in our overweight subjects because their leptin levels were not very hyperleptinemic. In addition, plasma leptin did not exhibit a significant association with FMD in our lean subjects. This could be due to their low leptin levels (2.5 ng/mL), at which leptin has not been found to exert significant vasodilation in Japanese subjects [10]. Second, forearm plethysmography reflects endothelial function of the resistance artery mediated mainly by the EDHF, whereas FMD reflects the conduit artery by NO [9]. An animal study showed that even if the endothelial NO synthase-derived NO production is impaired or absent, leptin can induce neuronal NO synthase in the endothelium to maintain endothelium dependent-vasorelaxation in a mouse model of obesity with hyperleptineima or angiotensin-II-induced vascular dysfunction [43]. Therefore, in this study, NO-mediated vasodilation assessed with FMD of the brachial artery was observed in overweight diabetic subjects with mildly elevated plasma leptin levels. Third, overweight subjects in this study were significantly younger than the lean subjects. Endothelial dysfunction assessed by FMD is recognized as an early marker of vascular damage, contributing to the initiation and progression of atherosclerosis [7]. Although intima-media thickness of the carotid artery did not significantly differ between groups (lean, 1.06 ± 0.60 mm; overweight, 0.99 ± 0.45 mm; *p* = 0.456), it could be speculated that the overweight subjects in our study had less advanced atherosclerosis, and were thus able to respond to the vasodilator effect of moderately elevated plasma leptin levels at the time of FMD measurement.

Our results further demonstrated that plasma leptin levels were also independently and positively associated with NMD in both lean and overweight subjects and those associations were found only after adjusting for other confounders including age, obesity, BP, and lipids. The vasodilator response to exogenous NO reflects vascular smooth muscle function and is reported to be impaired independently of endothelial dysfunction in subjects at risk for atherosclerosis [26]. Apart from the endothelium, leptin was also shown to directly target vascular smooth muscle cells via NO-dependent [51] and NO/endothelium independent [44] manner. Thus, the correlation between leptin and NMD in both lean and overweight subjects may reflect the smooth muscle-dependent vasodilator effect of leptin, which can be observed even in lean T2D patients with low plasma leptin levels.

There were a few limitations of our study. First, this was a cross-sectional study; therefore, a causal relationship between plasma leptin and FMD cannot be clarified. Second, the patients with T2D in this study were receiving various anti-atherogenic drug interventions such as anti-hypertensive agents, statins and insulin therapy that can exert considerable effects on FMD of the brachial artery and related atherosclerotic risk factors. To minimize the effect of such treatments, we adjusted for patient treatment status in our multivariate analyses. Third, our overweight subjects were significantly younger than the lean subjects. A positive relationship between BMI and FMD was also found in the univariate analysis. Since all potential confounding risk factors could not be adjusted for with the consecutive inclusion of our subjects, factors including age and BMI were adjusted for and the independent association of leptin was confirmed in the multivariate analyses. Fourth, no healthy controls were used to compare our findings, and we could not confirm that FMD was impaired in our study population of T2D patients. Last, this study included a very low number of morbidly obese patients with a BMI ≥ 30 kg/m^2^ (n = 15, 8.8%); thus, our results are only applicable to normal or overweight T2D patients. Leptin could contribute differently to FMD in morbidly obese patients with more severe leptin resistance and hyperleptinemia than overweight subjects [15,49,52].

Further studies with a larger population that includes T2D patients with a wide range of BMI are required to validate these findings. Furthermore, prospective and interventional studies assessing changes in both plasma leptin levels and FMD are warranted to clarify whether plasma leptin levels are predictive of vascular endothelial function in patients with obesity and T2D.

## Conclusions

Our data demonstrate that the plasma leptin level is an independent determinant of better FMD of the brachial artery in overweight, but not lean, patients with T2D. The present study provides clinical evidence that leptin is associated with vascular endothelial function in T2D patients with moderate obesity.

## Abbreviations

BMI: Body mass index; FMD: Flow-mediated dilatation; NO: Nitric oxide; MetS: Metabolic syndrome; EDV: Endothelium-dependent vasodilatation; T2D: Type 2 diabetes; SBP: Systolic blood pressure; NMD: Endothelium-independent nitroglycerin-mediated dilatation; SD: Standard deviation; LDL-C: Low-density lipoprotein cholesterol; ARB: ASngiotensin-II receptor blockers; ACEI: Angiotensin-converting enzyme inhibitors; HDL-C: High-density lipoprotein cholesterol; EDHF: Endothelium-derived hyperpolarizing factor.

## Competing interests

The authors declare that they have no competing interest.

## Authors’ contributions

TM and ME conceived of the study, participated in its design and coordination, and helped to draft the manuscript. TM carried out the immunoassays, and performed the statistical analysis. YY, NK, SI, RN, and HU recruited patients and carried out the vascular ultrasound. KMot, KMor, SF, HK, TS and MI were involved in drafting the manuscript or revising it critically. All authors read and approved the final manuscript.
